# Association between Life's Essential 8 score and high‐sensitivity C‐reactive protein: A cross‐sectional study from NHANES 2015−2018

**DOI:** 10.1002/clc.24270

**Published:** 2024-04-16

**Authors:** Jianan Li, Jie Zhang, Dan Su, Sanru Lin, Yujie Huang, Shujing Wu, Demin Xu

**Affiliations:** ^1^ School of Public Health Xiamen University Xiamen Fujian China; ^2^ Medical Department, Zhongshan Hospital (Xiamen branch) Fudan University Xiamen Fujian China; ^3^ Department of Cardiology, Zhongshan Hospital (Xiamen branch) Fudan University Xiamen Fujian China; ^4^ Department of Cardiac Surgery, Zhongshan Hospital Fudan University Shanghai China

**Keywords:** cardiovascular health, high‐sensitivity C‐reactive protein, inflammatory biomarker, life's essential 8

## Abstract

**Background:**

Earlier studies showed a negative correlation between life's simple 7 (LS7) and high‐sensitivity C‐reactive protein (hs‐CRP), but no association has been found between life's essential 8 (LE8), an improved version of LS7, and hs‐CRP.

**Hypothesis:**

This study investigated the association between LE8 and hs‐CRP utilizing data from the National Health and Nutritional Examination Survey.

**Methods:**

A total of 7229 adults were incorporated in our study. LE8 was scored according to American Heart Association guidelines, and LE8 was divided into health behaviors and health factors. Serum samples of the participants were used to measure hs‐CRP. To investigate the association between LE8 and hs‐CRP, weighted linear regression, and restricted cubic spline were utilized.

**Results:**

Among 7229 participants, the average age was 48.03 ± 16.88 years, 3689 (51.2%) were females and the median hs‐CRP was 1.92 (0.81−4.49) mg/L. In adjusted weighted linear regression, a negative correlation was observed between the LE8 score and hs‐CRP. Compared with the low LE8 score, the moderate LE8 score *β* was −0.533 (−0.646 to −0.420), and the high LE8 score *β* was −1.237 (−1.376 to −1.097). Health behaviors and health factors were also negatively associated with hs‐CRP. In stratified analyses, the negative correlation between LE8 and hs‐CRP remained consistent across subgroups.

**Conclusion:**

There was a negative correlation between LE8 as well as its sub‐indicator scores and hs‐CRP. Maintaining a positive LE8 score may be conducive to lowering the level of hs‐CRP.

## Graphical abstract

The results of this study showed a negative correlation between life's essential 8 (LE8) and high‐sensitivity C‐reactive protein (hs‐CRP), and a stronger association between health factors and hs‐CRP than health behaviors.

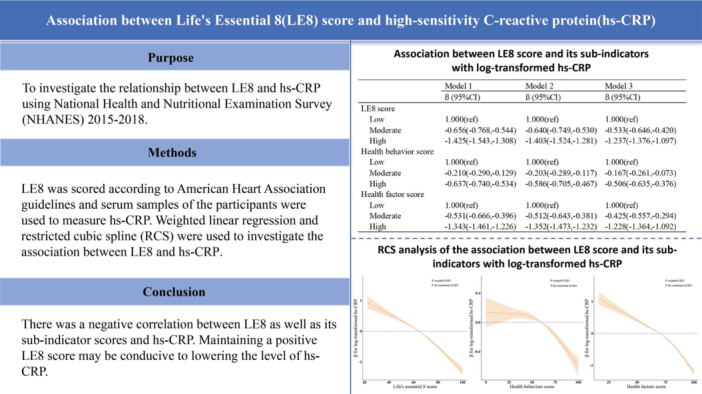

## 1 INTRODUCTION

To promote the cardiovascular health (CVH) in American adults, the American Heart Association (AHA) proposed life's simple 7 (LS7) in 2010 and life's essential 8 (LE8) in 2022.^1^ As an updated metric based on LS7, LE8 added a sleep metric and proposed the notion of enhancing CVH across the lifespan.^2^

High‐sensitivity C‐reactive protein (hs‐CRP) has been used as a biomarker of cardiovascular disease (CVD) prognosis.^3^, ^4^ High concentrations of hs‐CRP are related to the presence of CVD, such as coronary atherosclerosis, atrial fibrillation, and stroke.^5^, ^6^, ^7^ Earlier studies have shown an inverse association between LS7 scores and CVD biomarkers such as aldosterone, interleukin‐6, and CRP.^8^, ^9^, ^10^ But there are fewer articles on the relationship between LE8 and hs‐CRP. The purpose of our study is to detect the relationship between LE8 and hs‐CRP using the National Health and Nutritional Examination Survey (NHANES) database from 2015 to 2018.

## 2 METHODS

### 2.1 Study population

**Figure 1:**
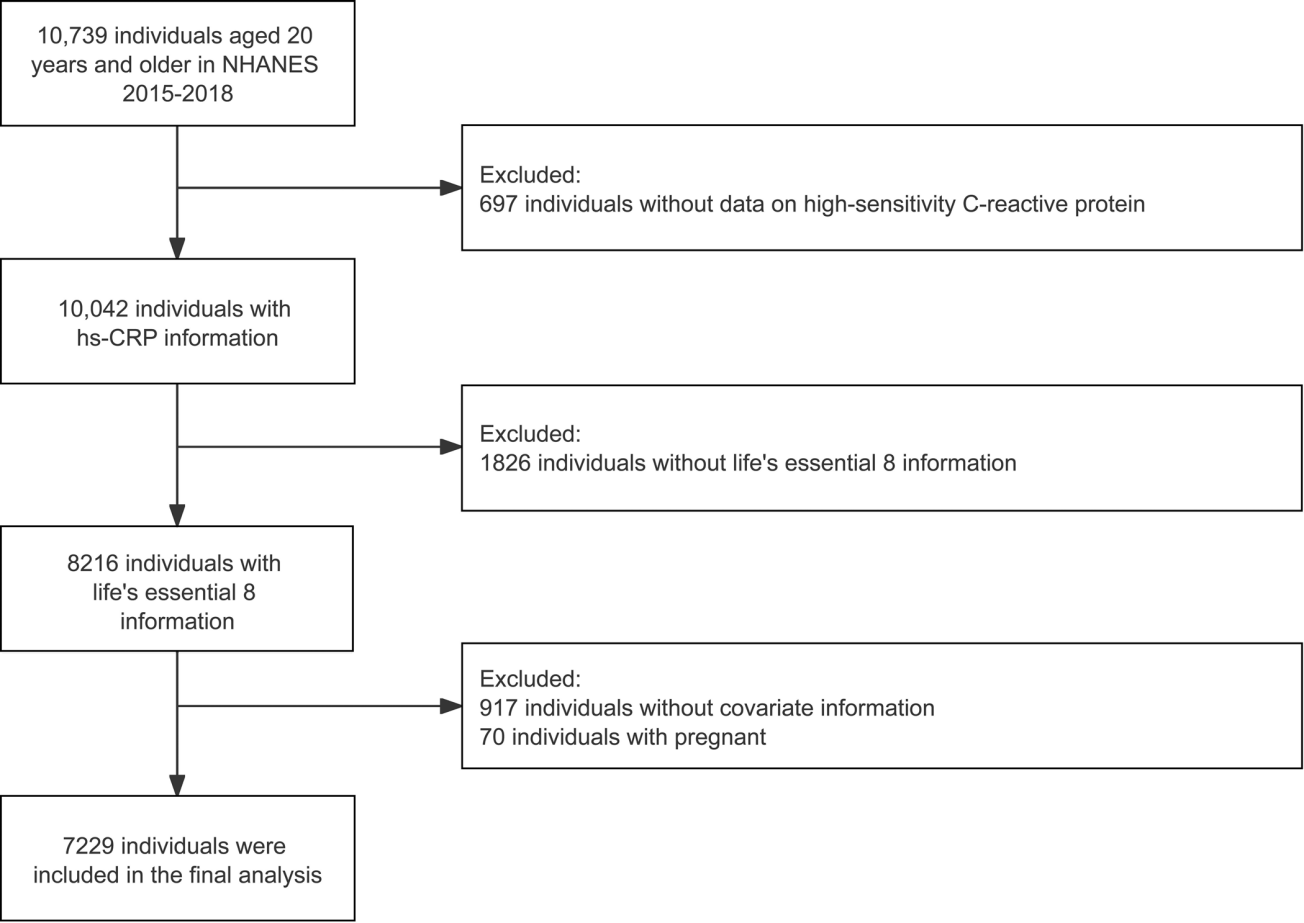
Flowchart of selecting participants in NHANES 2015−2018. NHANES, National Health and Nutritional Examination Survey.

A cross‐sectional survey using complex multistage probability sampling, NHANES, is designed to obtain a nationally representative sample of adults and children in the United States. Our study used data from two consecutive cycles in NHANES from 2015 to 2018. All participants signed informed consent. Among the 10,739 adults aged ≥20 years, we excluded (1) subjects missing hs‐CRP results (*n* = 697), (2) subjects missing LE8 score indicators (*n* = 1826), (3) subjects missing potential covariates (*n* = 917), and (4) subjects who were already pregnant (*n* = 70). Ultimately, 7229 participants were selected as part of this survey (Figure 1).

### 2.2 Definition of hs‐CRP

Participants provided serum samples at the time of the survey, which were stored under adequate refrigeration conditions (−30°C) and then sent to the cooperating laboratory for examination. Specific sample handling and laboratory testing methods have been posted on the official NHANES website.^11^ Due to the existence of a lower limit of detection (LLOD) for the hs‐CRP assay instrument, results below the LLOD were estimated using the LLOD divided by the square root of 2 (LLOD/sqrt [2]) according to the relevant NHANES instructions. In our study, a total of 190 individuals had hs‐CRP values below the LLOD, and all of these individuals were analyzed using estimated LLOD values.

### 2.3 Definitions of LE8

Four ideal health behaviors (dietary patterns, physical exercise, nicotine exposure, and sleep duration) and four ideal health factors (body mass index [BMI], blood lipids, blood glucose, and blood pressure) comprise the LE8 score. The dietary metric was measured by the Healthy Eating Index 2015 and combined with data from the study participants' first day 24 h dietary review. Self‐reported questionnaires were utilized to measure physical exercise, nicotine exposure, and sleep duration. BMI was calculated by weight divided by height squared (kg/m^2^), while blood pressure was determined from the average of three measurements. Blood samples were collected for lipid and glucose measurement. Blood lipids were measured via total cholesterol minus high‐density lipoprotein cholesterol, and blood glucose was measured by combining a self‐reported history of diabetes with glycated hemoglobin results. The score for each LE8 metric ranged from 0 to 100, and the overall LE8 score for participants was the mean of the eight metrics. According to the recommendations of the AHA, we classified the LE8 score into three levels: low (0−49), moderate (50−79), and high (80−100), while the sub‐indicators of LE8 were also classified into low, moderate, and high categories.^12^

### 2.4 Covariates

In our study, covariates included age, sex (male and female), race (Mexican American, non‐Hispanic Black, non‐Hispanic White, other Hispanic, and other race), marital status (married/living with a partner, divorced/separated/widowed, never married), education level (below high school, high school, above high school), poverty‐to‐income ratio (PIR) (<1.3, 1.3−3.5, >3.5), hypertension, diabetes, cancer, and CVD. Cancer and CVD were identified by self‐report, while hypertension and diabetes were identified by self‐report and laboratory measurements.^13^

### 2.5 Statistical analysis

The NHANES utilized complex multistage sampling to collect baseline data. To ensure national representation, weighting is required in the statistical analysis of NHANES data. When characterizing baseline information, continuous variables were described using weighted mean ± standard deviation, and categorical variables were described using sample size (weighted percentages). One‐way analysis of variance was utilized to describe the relationship between continuous variables and LE8 scores. The Rao−Scott χ^2^ test was utilized to describe the association between categorical variables and LE8 scores. Given the skewed distribution of the raw hs‐CRP data, we used log‐transformed hs‐CRP for statistical analyses.

Weighted linear regression was applied to reflect the relationship between LE8 and its sub‐indicators with log‐transformed hs‐CRP. In the regression model, LE8 and its sub‐indicators were analyzed as categorical variables (low, moderate, and high) as well as continuous variables (every 10‐point increment). *β* Values with 95% confidence intervals were utilized to reflect the association between the two variables. Regression models were adjusted for potential confounders, including age, sex, race, marital status, education level, PIR, CVD, hypertension, cancer, and diabetes.

Subgroup analyses were performed to analyze the relationship between LE8 score and log‐transformed hs‐CRP in different subgroup populations. Analyses were stratified by age, sex, race, marital status, education level, PIR, CVD, hypertension, cancer, and diabetes. A multiplicative interaction test was used to calculate potential interactions between stratified covariates and LE8 score. In addition, nonlinear associations between LE8, health behaviors, and health factors with log‐transformed hs‐CRP were analyzed by restricted cubic spline models (RCS). Weighted linear models of RCS were modified according to age, sex, race, marital status, education level, PIR, CVD, hypertension, cancer, and diabetes. All data analysis was done through R software (version 4.3.1). Two‐sided tests were used for all statistical analyses, and *p* < .05 was deemed statistically significant.

## 3 RESULTS

### 3.1 Baseline characteristics of the study population

Baseline features of individuals grouped by LE8 score were displayed in Table 1. Among the 7229 individuals, the average age was 48.03 ± 16.88, with 3689 (51.2%) females and a majority of non‐Hispanic whites (66.8%). Regarding marital status and education level, 4418 (65.0%) of the study participants were married or living with others, and 4131 (64.6%) were at or above the high school level of education. In terms of demographic characteristics, participants with higher LE8 score were younger, more probably female, with more stable marital status, higher levels of education, and higher incomes compared to those with lower LE8 score. For chronic disease, individuals with higher LE8 score were less susceptible to hypertension, diabetes, cancer, and CVD than those with lower LE8 score. Among all participants, the median hs‐CRP level was 1.92 (0.81−4.49), and those with a high LE8 score had lower hs‐CRP level compared to those with a low LE8 score.

**Table 1:** Features of baseline information of study participants grouped according to LE8 score.

	LE8 score				*p* Value
Characteristic	Total	Low	Moderate	High	
*N*	7229	1152	4821	1256	
Age	48.03 ± 16.88	51.58 ± 14.82	49.23 ± 17.04	42.38 ± 16.23	<.001
Sex					<.001
Male	3540 (48.8)	623 (54.0)	2404 (50.5)	513 (40.7)	
Female	3689 (51.2)	529 (46.0)	2417 (49.5)	743 (59.3)	
Race					<.001
Mexican American	1119 (8.4)	208 (11.4)	757 (8.4)	154 (6.8)	
Other Hispanic	814 (6.0)	119 (5.5)	561 (6.1)	134 (5.9)	
Non‐Hispanic White	2675 (66.8)	421 (62.9)	1769 (66.4)	485 (70.4)	
Non‐Hispanic Black	1487 (9.8)	297 (13.5)	1026 (10.4)	164 (5.9)	
Other race	1134 (8.9)	107 (6.8)	708 (8.7)	319 (10.9)	
Marital status					<.001
Married/living with a partner	4418 (65.0)	637 (60.0)	2991 (65.2)	790 (67.7)	
Divorced/separated/widowed	1533 (17.5)	342 (24.8)	1047 (18.4)	144 (10.2)	
Never married	1278 (17.5)	173 (15.2)	783 (16.4)	322 (22.1)	
Education level					<.001
Below high school	1419 (11.2)	323 (18.7)	994 (12.2)	102 (3.7)	
High school	1679 (24.2)	331 (35.4)	1154 (25.6)	194 (13.4)	
Above high school	4131 (64.6)	498 (45.9)	2673 (62.2)	960 (83.0)	
PIR					<.001
<1.3	2116 (19.3)	453 (30.1)	1415 (19.5)	248 (12.4)	
1.3−3.5	2953 (36.5)	513 (43.9)	2004 (37.1)	436 (30.4)	
>3.5	2160 (44.2)	186 (26.1)	1402 (43.4)	572 (57.1)	
Previous history of CVD	778 (8.2)	207 (15.6)	530 (8.3)	41 (3.3)	<.001
Cancer	703 (10.8)	126 (10.8)	499 (11.9)	78 (7.6)	.002
Hypertension	3108 (37.4)	758 (63.9)	2160 (40.2)	190 (13.3)	<.001
Diabetes	1280 (12.5)	445 (32.5)	802 (12.2)	33 (1.5)	<.001
hs‐CRP, median (IQR)	1.92 (0.81−4.49)	3.72 (1.90−7.24)	1.99 (0.90−4.40)	0.87 (0.46−2.00)	<.001

Abbreviations: CVD, cardiovascular disease; hs‐CRP, high‐sensitivity C‐reactive protein; IQR, interquartile range (P25−P75); LE8, life's essential 8; PIR, poverty‐to‐income ratio.

### 3.2 Relationship between LE8 and its sub‐indicators with log‐transformed hs‐CRP

We analyzed the association between the LE8 score and its sub‐indicators with hs‐CRP by weighted linear regression (Table 2). In model 3, the adjusted *β* of hs‐CRP was −0.317 (95% CI: −0.341 to −0.293) per 10‐point increase in LE8 score. In contrast to individuals with low LE8 score, the adjusted *β* for hs‐CRP was −0.533 (95% CI: −0.646 to −0.420) for individuals with moderate LE8 score and −1.237 (95% CI: −1.376 to −1.097) for individuals with high LE8 score.

**Table 2:** Relationship between LE8 score and its sub‐indicators with log‐transformed hs‐CRP.

	Model 1	Model 2	Model 3
	*β* (95% CI)	*β* (95% CI)	*β* (95% CI)
LE8 score (continuous) per 10 points increase	−0.317 (−0.341 to −0.293)	−0.326 (−0.352 to −0.301)	−0.300 (−0.330 to −0.270)
LE8 score			
Low	1.000 (ref)	1.000 (ref)	1.000 (ref)
Moderate	−0.656 (−0.768 to −0.544)	−0.640 (−0.749 to −0.530)	−0.533 (−0.646 to −0.420)
High	−1.425 (−1.543 to −1.308)	−1.403 (−1.524 to −1.281)	−1.237 (−1.376 to −1.097)
Health behavior score (continuous) per 10 points increase	−0.114 (−0.131 to −0.096)	−0.106 (−0.125 to −0.087)	−0.092 (−0.112 to −0.072)
Health behavior score			
Low	1.000 (ref)	1.000 (ref)	1.000 (ref)
Moderate	−0.210 (−0.290 to −0.129)	−0.203 (−0.289 to −0.117)	−0.167 (−0.261 to −0.073)
High	−0.637 (−0.740 to −0.534)	−0.586 (−0.705 to −0.467)	−0.506 (−0.635 to −0.376)
Health factor score (continuous) per 10 points increase	−0.284 (−0.306 to −0.262)	−0.297 (−0.321 to −0.273)	−0.292 (−0.319 to −0.265)
Health factor score			
Low	1.000 (ref)	1.000 (ref)	1.000 (ref)
Moderate	−0.531 (−0.666 to −0.396)	−0.512 (−0.643 to −0.381)	−0.425 (−0.557 to −0.294)
High	−1.343 (−1.461 to −1.226)	−1.352 (−1.473 to −1.232)	−1.228 (−1.364 to −1.092)

*Note*: *p* Values <.001 for all statistical analyses in this table.

Abbreviations: CVD, cardiovascular disease; hs‐CRP, high‐sensitivity C‐reactive protein; LE8, life's essential 8.

Model 1: Unadjusted covariates.

Model 2: Adjusted with age + sex + race + marital status + education level + PIR.

Model 3: Adjusted with hypertension + diabetes + cancer + CVD + Model 2.

Submetrics of LE8 were similarly negatively correlated with hs‐CRP. In Model 3, the adjusted *β* of hs‐CRP was −0.092 (95% CI: −0.112 to −0.072) for every 10‐point increase in health behavior score. Compared with participants with low health behavior score, hs‐CRP adjusted *β* was −0.167 (95% CI: −0.261 to −0.073) for participants with moderate health behavior score and −0.506 (95% CI: −0.635 to −0.376) for participants with high health behavior score. Similarly, the adjusted *β* of hs‐CRP was −0.292 (95% CI: −0.319 to −0.265) for every 10‐point increase in health factor score. Compared with participants with low health factor score, the hs‐CRP adjusted *β* was −0.425 (95% CI: −0.557 to −0.294) for participants with moderate health factor score and −1.228 (95% CI: −1.364 to −1.092) for participants with high health factor score.

### 3.3 Relationship between sleep score and hs‐CRP

As sleep was the only variable that differed between LS7 and LE8, we analyzed the association between sleep score and hs‐crp. The association between sleep score and hs‐CRP was analyzed by weighted linear regression (Supporting Information S1: Table 1). In the adjusted model, *β* for hs‐CRP was −0.009 (95% CI: −0.027 to 0.009) for each 10‐point increase of sleep score.

### 3.4 Subgroup analysis

**Figure 2:**
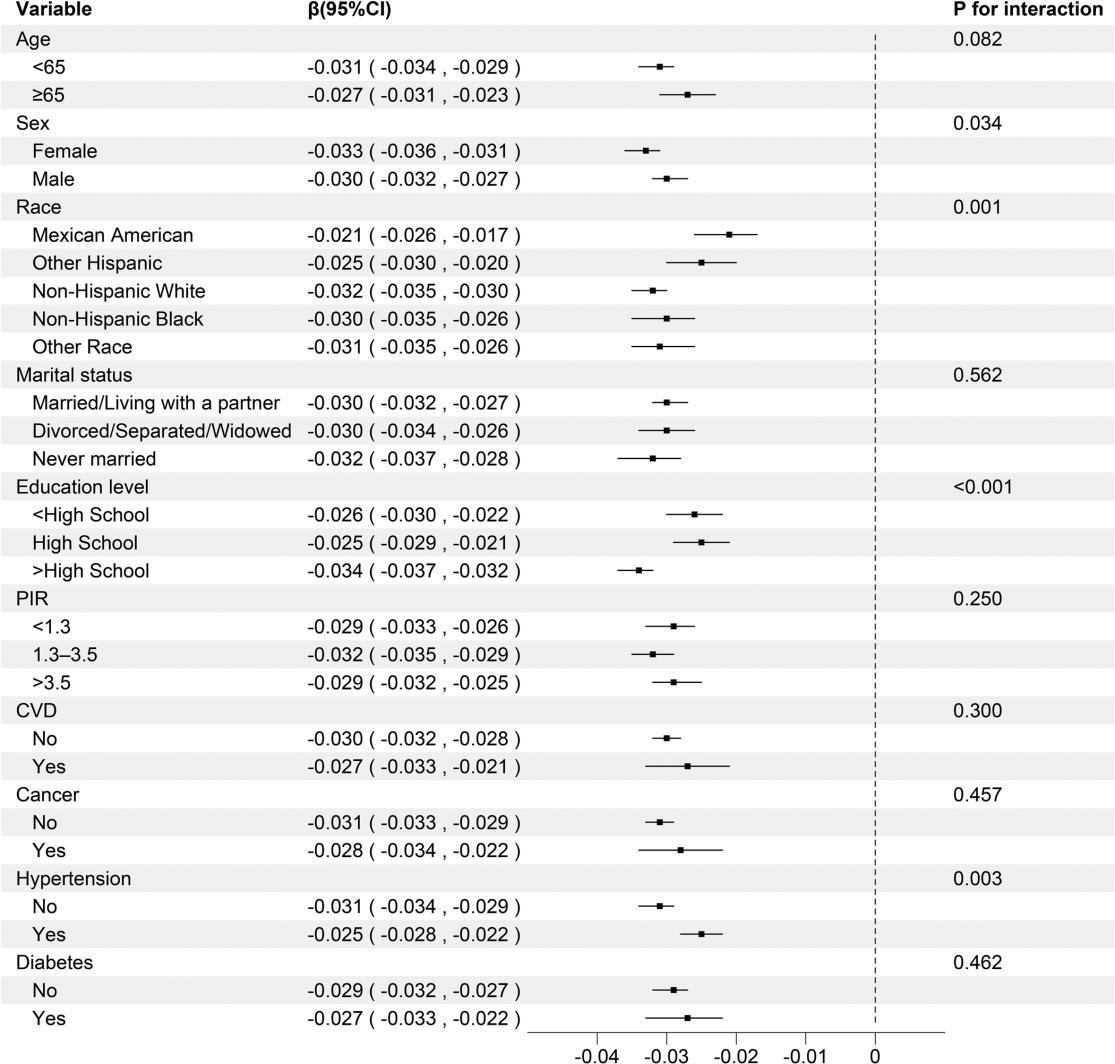
Subgroup analysis of the relationship between LE8 and log‐transformed hs‐CRP. CVD, cardiovascular disease; hs‐CRP, high‐sensitivity C‐reactive protein; LE8, life's essential 8; PIR, poverty‐to‐income ratio.

Subgroup analysis results were displayed in Figure 2. After stratification by covariates, LE8 and hs‐CRP negatively correlated among the subgroup variables. This negative correlation remained consistent with the overall analysis results.

### 3.5 RCS analysis

**Figure 3:**
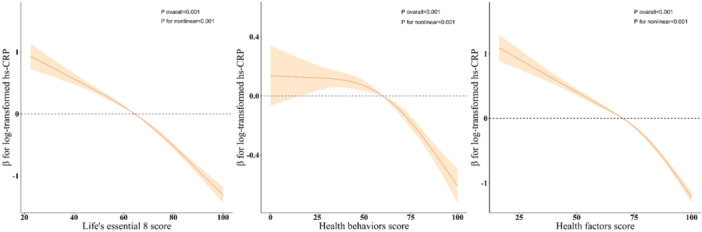
RCS analysis of the association among LE8 score, health behavior score, and health factor score with hs‐CRP. Age, sex, race, marital status, PIR, education level, hypertension, diabetes, CVD, and cancer were adjusted in the RCS models. CVD, cardiovascular disease; hs‐CRP, high‐sensitivity C‐reactive protein; LE8, life's essential 8; PIR, poverty‐to‐income ratio; RCS, restricted cubic spline.

Nonlinear associations of LE8 and its sub‐indicators with hs‐CRP were analyzed by RCS regression (Figure 3). The adjusted RCS plots showed a nonlinear association between LE8 and its sub‐indicators with hs‐CRP (*p* for nonlinear <.05).

## 4 DISCUSSION

We conducted an analysis of 7229 individuals from NHANES to clarify the association between LE8 and hs‐CRP. The findings indicate a negative association between LE8 and its sub‐indicators with hs‐CRP. Furthermore, this association remained stable across subgroups, including age, sex, race, marriage, education, income, hypertension, diabetes, CVD, and cancer. Interestingly, there was a nonlinear relationship between LE8 and its sub‐indicators with hs‐CRP.

Previous studies have shown that a higher score of LS7 is closely associated with a lower level of CVD inflammatory markers.^14^, ^15^, ^16^ Results from a longitudinal study of 6814 individuals suggested that higher LS7 scores were related to lower levels of multiple CVD biomarkers such as hs‐CRP, d‐dimer, and interleukin‐6.^14^ A study from China found a negative association between LS7 score and biomarkers of CVD, including CRP, d‐dimer, and interleukin‐6.^15^ Results from Framingham cohort study suggested that the negative association between the ideal LS7 score and CVD incidence was partly related to CVD biomarker levels, and that high CVH scores were associated with low biomarker levels.^16^ In contrast to previous studies, we used a novel LE8 metric and found that there was a negative relationship between the LE8 score and hs‐CRP. This negative association was consistent with the findings of earlier LS7 studies, suggesting that increasing both LS7 score and LE8 score may reduce the level of hs‐CRP.

In our study, the association between health factors and hs‐CRP was stronger than health behaviors. Results from a longitudinal study of 6814 participants showed that the association of BMI with hs‐CRP was significantly stronger than other metrics.^14^ BMI also had a stronger association with hs‐CRP than other LE8 metrics in our study (Supporting Information S1: Table 1). Therefore, it may be the BMI that contributed to the stronger association of health factors with hs‐CRP than health behaviors.

Previous studies indicated that sleep duration was associated with blood levels of hs‐CRP.^17^, ^18^, ^19^ A cohort study from UK Biobank showed that participants who slept 7−8 h per day had the lowest hs‐CRP level and that less than 7 h or more than 8 h had higher hs‐CRP level.^18^ In our study, 7−9 h of sleep per day obtained the highest sleep score, and too little or too much sleep duration obtained a lower sleep score. So there was a negative correlation between sleep score and hs‐CRP in our study, which is consistent with previous findings.

The blood level of hs‐CRP is associated with a variety of factors. It has been shown that a higher frequency of vegetable or legume intake in the diet is negatively correlated with hs‐CRP level.^20^ In contrast, poor lifestyles such as sleep deprivation, persistent smoking, and lack of physical activity increase hs‐CRP level in the body.^21^, ^22^, ^23^ Additionally, it has also been demonstrated that hs‐CRP level was generally higher in those who were obese, dyslipidemic, and suffer from hypertension.^24^, ^25^, ^26^ Therefore, the LE8 score, as a comprehensive indicator combining the above eight indicators, may be related to serum hs‐CRP level.

There are several limitations in our study. First, our study was cross‐sectional and could not make inferences at the level of a causal relationship between LE8 and hs‐CRP. Second, when calculating the LE8 score, self‐reported data from participants were used for many indicators, and there may be recall bias in these data. Finally, it was difficult for us to conduct further studies because of the absence of data on other cardiovascular biomarkers in the NHANES 2015–2018 data set.

In summary, there was a negative correlation between LE8 and its sub‐indicators with hs‐CRP. Maintaining an optimal LE8 score was connected with lower concentrations of hs‐CRP. Further clinical studies are required to clarify the inherent mechanisms between LE8, hs‐CRP, and the occurrence of CVD.

## AUTHOR CONTRIBUTIONS

Jianan Li conceived the study and drafted the manuscript. Jianan Li and Jie Zhang analyzed the data. Dan Su, Sanru Lin, and Yujie Huang verified the data. Shujing Wu and Demin Xu revised the manuscript. All authors contributed to the article and approved the submitted version.

## CONFLICT OF INTEREST STATEMENT

The authors declare no conflict of interest.

## Supporting information

Supporting information.

## Data Availability

All data sets of this study are available on the National Health and Nutrition Examination Survey website (https://www.cdc.gov/nchs/nhanes).
