# Supplementary material for: Association between Life's Essential 8 score and high‐sensitivity C‐reactive protein: A cross‐sectional study from NHANES 2015−2018
Source: Clin Cardiol. 2024 Apr 16;47(4):e24270. doi: 10.1002/clc.24270 (PMC11021857; doi:10.1002/clc.24270)
Supplement: Supplementary file 1 — Supporting information. [file CLC-47-e24270-s001.docx]

**Association between Life's Essential 8 score and high-sensitivity C-reactive protein：A cross-sectional study from NHANES 2015-2018**

**Online Supplement**

Jianan Li ^1^, Jie Zhang ^1^, Dan Su ^1^, Sanru Lin ^1^, Yujie Huang ^2^, Shujing Wu ^3^, Demin Xu^2,4^

Jianan Li and Jie Zhang contributed equally to this work and share first authorship.

**Author Affiliations:**

1 School of Public Health, Xiamen University, Xiamen, Fujian, China

2 Medical Department, Zhongshan Hospital (Xiamen), Fudan University, Xiamen, Fujian, China

3 Department of Cardiology, Zhongshan Hospital (Xiamen), Fudan University, Xiamen, Fujian, China

4 Department of Cardiac Surgery, Zhongshan Hospital, Fudan University, Shanghai, China

**Table S1** Relationship between each LE8 metric and log-transformed hs-CRP

| LE8 metrics (per 10 points increase) | Model 1  β (95%CI) | Model 2  β (95%CI) | Model 3  β (95%CI) |
| --- | --- | --- | --- |
| Diet score | -0.055 (-0.067, -0.044) | -0.065 (-0.077, -0.053) | -0.058 (-0.071, -0.045) |
| Physical activity score | -0.046 (-0.056, -0.036) | -0.043 (-0.052, -0.033) | -0.036 (-0.046, -0.026) |
| Tobacco exposure score | -0.024 (-0.033, -0.014) | -0.030 (-0.039, -0.021) | -0.023 (-0.033, -0.014) |
| Sleep health score | -0.022 (-0.037, -0.007) | -0.023 (-0.038, -0.008) | -0.009 (-0.027, 0.009) |
| Body mass index score | -0.172 (-0.184, -0.160) | -0.170 (-0.182, -0.159) | -0.168 (-0.181, -0.155) |
| Blood lipids score | -0.061 (-0.073, -0.049) | -0.061 (-0.073, -0.049) | -0.059 (-0.072, -0.047) |
| Blood glucose score | -0.110 (-0.124, -0.096) | -0.112 (-0.128, -0.097) | -0.106 (-0.122, -0.091) |
| Blood pressure score | -0.066 (-0.083, -0.050) | -0.064 (-0.081, -0.048) | -0.058 (-0.074, -0.041) |

Model 1: unadjusted covariates

Model 2: adjusted with age and sex

Model 3: adjusted with age, sex, race, marital status, education level, and PIR.
